# Evaluation of Hemodialysis Efficacy for Serum and Salivary Clearance of Urea, Creatinine, and Uric Acid in Chronic Kidney Disease Patients: Short title: Evaluation of Hemodialysis Efficacy in CKD Patients

**DOI:** 10.31661/gmj.v15i.4098

**Published:** 2026-05-04

**Authors:** Azam Hosseini, Farid Abbasi, Iraj Mirzaii-Dizgah, Reza Afshar

**Affiliations:** ^1^ Department of Oral and Maxillofacial Medicine, Faculty of Dentistry, Shahed University, Tehran, Iran; ^2^ Department of Physiology, AJA University of Medical Science, Tehran, Iran; ^3^ Department of Nephrology, Mostafa Khomeini Hospital, Shahed University, Tehran, Iran

**Keywords:** Hemodialysis, Saliva, Urea, Creatinine, Uric Acid

## Abstract

**Background:**

Chronic kidney disease (CKD) is a progressive and irreversible loss of kidney function that may progress to end-stage renal disease requiring hemodialysis. While hemodialysis is an established life-saving therapy, simple methods to assess its adequacy are needed. Saliva analysis is a non-invasive alternative to serum testing. This study evaluated changes in urea, creatinine, and uric acid levels in serum before and after hemodialysis and compared them with salivary levels to assess the adequacy of hemodialysis.

**Materials and Methods:**

A case-control study was conducted on 32 patients undergoing hemodialysis and 30 healthy controls. Blood and saliva samples were collected immediately before and after dialysis from patients and controls. Serum and salivary levels of markers were analyzed by biochemical methods. Serum and salivary levels of markers were compared between the patient group (before and after dialysis) and the control group by t-test and the diagnostic accuracy of salivary markers compared to serum markers was evaluated by the receiver-operating characteristic (ROC) curve and area under the curve (AUC).

**Results:**

There were significant differences between the serum and salivary levels of urea, creatinine, and uric acid in patients before and after hemodialysis, and also with the control group (P0.05). There was a positive correlation between the serum and salivary levels of urea, creatinine and uric acid before hemodialysis, and creatinine after hemodialysis. The AUC for salivary urea and creatinine was 0.87 and 0.88, respectively, and the cut-off point for urea and creatinine was 55 mg/dL and 0.5 mg/dL, respectively. The ROC analysis for uric acid was not significant.

**Conclusion:**

This study showed that before hemodialysis, the changes in salivary urea, creatinine, and uric acid in CKD patients (in the fasting state) were similar to serum. Additionally, after hemodialysis, changes in salivary creatinine levels were similar to serum. Therefore, saliva analysis is suggested as a non-invasive alternative to serum analysis for assessment of the efficacy of hemodialysis.

## Introduction

Chronic kidney disease (CKD) is defined as a progressive and irreversible decline in kidney function (i.e., glomerular filtration rate [GFR]<60 mL/min/1.73 m2 or albuminuria >30 mg/24 hours) for 3 months or more [1].

CKD affects 8% to 16% of the world’s population [2]. The most common causes of CKD include diabetes mellitus (44%), followed by hypertension (28%), and chronic glomerulonephritis (16%) [3]. As the disease progresses and GFR decreases to less than 15 mL/min/1.73 m2, retention of metabolic products such as urea, creatinine, and uric acid, as well as changes in electrolyte balance of the serum occur. In these conditions, renal replacement therapy or hemodialysis must be performed to save the patient’s life. Effective hemodialysis as a life-saving treatment can help maintain homeostasis, and improve the quality of life of patients [4]. In such patients, serum analysis of chemical biomarkers and electrolytes is performed for diagnosis, monitoring of treatment outcomes, determination of prognosis, and evaluation of hemodialysis efficacy [1][5].

Drawing blood for serum analysis is an invasive procedure that is associated with pain and anxiety, as well as an increased risk of developing hepatitis C and B [1]. In addition, these patients are anemic for various reasons such as reduced erythropoietin production, abnormal iron metabolism, platelet and vascular changes, frequent blood draws, and blood loss in dialysis tubes [5]. It is noteworthy that each patient loses 4-20 mL of blood during the hemodialysis process [1]. Therefore, presence of a suitable alternative to blood that is reliable for diagnosis and monitoring of the disease is highly desirable for both patients and clinicians.

Saliva, as a biological fluid, plays an important role in systemic and oral health. Saliva collection is simple, non-invasive, and repeatable, and does not require medical personnel compared to serum collection. In addition, the risk of infection is low in this method [6].

The present study aimed to assess biochemical markers namely urea, creatinine, and uric acid in serum and saliva before and after hemodialysis to evaluate the efficacy of hemodialysis in CKD patients.

## Materials and Methods

This investigation was conducted as a hospital-based case-control study designed to evaluate the association between salivary and serum biochemical markers and the presence of CKD. Participants were selected based on disease status (CKD versus absence of CKD), and the distribution of biochemical marker levels was compared between the two groups.

### Study Population and Setting

The study population included 62 participants recruited from Shahid Mostafa Khomeini Hospital and Shahid Mostafa Chamran Hospital. The case group consisted of 32 patients with a confirmed diagnosis of chronic kidney disease who were undergoing maintenance hemodialysis. The control group included 30 apparently healthy individuals without a history or clinical evidence of kidney disease who presented to the same hospital for routine medical checkups. Recruiting both groups from the same source population was intended to reduce selection bias.

### Definition of Exposure and Outcome

The outcome of interest in this case-control study was the presence of chronic kidney disease. The exposure variables were defined as serum and salivary concentrations of urea, creatinine, and uric acid. Hemodialysis status and timing of sample collection (pre- and post-hemodialysis) were considered exposure-related conditions influencing biomarker levels among cases. The study compared the distribution of these exposures between cases and controls to assess their association with CKD.

### Sample Collection Procedures

For the case group, venous blood samples (5 mL) and unstimulated whole saliva samples (2 mL) were collected immediately before and immediately after hemodialysis sessions. Patients underwent hemodialysis three times per week, with each session lasting approximately three hours. Saliva samples were obtained using the spitting method to avoid stimulation-related variability. Patients were required to fast for at least eight hours prior to pre-dialysis sample collection; fasting was not required for post-dialysis sampling. For the control group, blood and saliva samples were collected once under fasting conditions using identical procedures. All samples were immediately transferred to the laboratory for analysis.

### Laboratory Measurements

Serum and salivary levels of urea, creatinine, and uric acid were measured using standardized biochemical methods. Urea concentration was determined using the Berthelot method, creatinine was measured using the Jaffe reaction, and uric acid levels were assessed using an enzymatic colorimetric method [7][8]. All assays were performed using commercially available kits supplied by Pars Azmoun Company (Karaj, Iran) in accordance with the manufacturer’s instructions.

### Statistical Analysis

Statistical analyses were performed using appropriate analytical software. Continuous variables were reported as mean ± standard error of the mean (SEM). Independent t-tests were used to compare biomarker levels between cases and controls, while paired t-tests were applied to compare pre- and post-hemodialysis values within the case group. Pearson correlation coefficients were calculated to assess the relationship between serum and salivary biomarker levels. Receiver-operating characteristic (ROC) curve analysis was conducted to evaluate the ability of salivary biomarkers to discriminate between CKD cases and controls, and optimal cut-off values were calculated to determine sensitivity, specificity, and area under the curve (AUC). A P-value less than 0.05 was considered statistically significant.

## Results

**Table:** Table1. Serum and Salivary Levels of Urea, Creatinine, and Uric Acid in CKD Patients (Before and After Hemodialysis) and the Control Group

**Parameter**	**Control **	**CKD (Before HD)**	**CKD (After HD)**	**P-value (Control vs. Before)**	**P-value (Before vs. After)**	**P-value (Control vs. After)**
**Urea (mg/dL)**						
Serum	31.6 ± 3.1	87.2 ± 7.8	31.5 ± 3.5	<0.001	<0.001	0.342
Saliva	51.5 ± 3.4	122.4 ± 10.3	57.5 ± 5.9	<0.001	<0.001	0.987
**Creatinine (mg/dL)**						
Serum	1.05 ± 0.03	8.48 ± 0.55	4.26 ± 0.34	<0.001	<0.001	0.002
Saliva	0.38 ± 0.03	2.75 ± 0.61	0.97 ± 0.17	<0.001	<0.001	<0.001
**Uric acid (mg/dL)**						
Serum	5.14 ± 0.29	8.03 ± 0.28	2.82 ± 0.14	<0.001	<0.001	<0.001
Saliva	3.48 ± 0.25	7.19 ± 1.22	2.19 ± 0.46	<0.001	<0.001	<0.001

**Table:** Table2. Diagnostic Accuracy of Salivary Biomarkers for CKD

**Biomarker**	**Salivary Cut-off**	**Sensitivity (%)**	**Specificity (%)**	**AUC**	**P value**
**Urea**	55 mg/dL	84	70	0.876	<0.001
**Creatinine**	0.5 mg/dL	81	94	0.881	<0.001
**Uric acid**	-	-	-	-	Not significant

AUC: Area under the ROC curve

**Figure-1:**
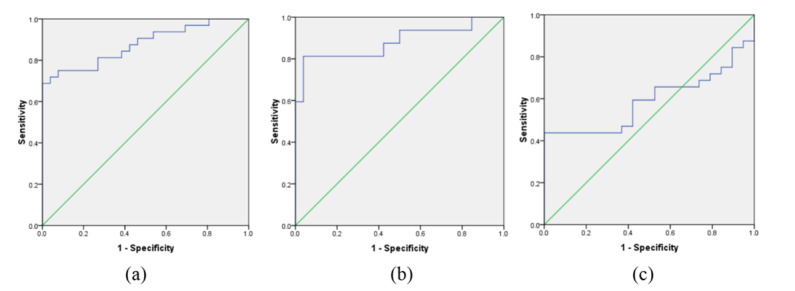
Figure 1. Area under curve in ROC analysis of salivary urea (a), creatinine (b), and uric acid (c) for CKD patients

The study included 32 patients (19 males, 59.4%; 13 females, 40.6%; mean age 59 ± 12 years) and 30 controls (15 males, 50.0%; 15 females, 50.0%; mean age 42 ± 10 years). There was no significant differences in gender distribution across the groups (P=0.48); While age was higher in CKD patients than healthy controls.

The mean serum and salivary levels of urea, creatinine, and uric acid in CKD patients (before and after hemodialysis) and in healthy controls are summarized in Table-1. Serum and salivary urea levels were significantly elevated in CKD patients before hemodialysis compared to both post-hemodialysis values and controls (P<0.001). Following hemodialysis, urea levels in serum and saliva decreased significantly and were comparable to those of healthy controls (P=0.342 and P=0.987, respectively).

Similarly, serum and salivary creatinine levels were markedly higher before hemodialysis than after hemodialysis and in controls (P<0.001). After hemodialysis, creatinine levels decreased significantly in both serum and saliva; however, they remained higher than in healthy controls (P=0.002 and P<0.001, respectively). There was a positive correlation between the serum and salivary levels of urea before hemodialysis (r=0.462, P=0.000), but there was no such correlation after hemodialysis (r=0.101, P=0.451). There was a positive correlation between the serum and salivary levels of creatinine before hemodialysis (r=0.621, P<0.001) and after hemodialysis (r=0.424, P=0.001). Also, the mean serum and salivary uric acid levels were significantly higher before hemodialysis than after hemodialysis and in healthy controls (P=0.000, Table-2). There was a positive correlation between the serum and salivary levels of uric acid before hemodialysis (r=0.307, P=0.045). However, there was no such correlation after hemodialysis (r=0.011, P=0.386).

To evaluate the diagnostic potential of salivary urea level compared to its serum level, ROC analysis was performed, which showed that the salivary urea cut-off point for CKD was 55 mg/dL with a sensitivity of 84%, specificity of 70%, and an AUC of 0.876 (Figure-1a). The salivary creatinine cut-off for CKD was 0.5 mg/dL with a sensitivity of 81%, specificity of 94%, and an AUC of 0.881 (Figure-1b); but ROC analysis was insignificant for uric acid levels (Figure-1c).

## Discussion

Urea, the end product of protein catabolism, is a small molecule that readily diffuses through biological membranes [4][9]. Normal salivary urea concentrations range from 12 to 70 mg/dL [5]. Creatinine is a muscle breakdown product that has a larger molecular weight and size than urea and therefore cannot easily pass through the salivary gland cells and intercellular junctions [4][10]. Normal salivary creatinine concentrations are 0.05 to 0.2 mg/dL [1][4]. Uric acid is a specific product of purine nucleotide catabolism that, due to its small size, freely passes from serum into the saliva [11][12]. Normal salivary uric acid concentrations range from 0.07 to 12 mmol/L [11].

Following CKD, the kidneys are unable to secrete these markers, and their concentration in the blood increases. This increased concentration gradient in the blood causes a change in permeability of the salivary glands and passive diffusion of these markers from serum to saliva. In other words, the body uses saliva as an excretory route during renal dysfunction [4][10].

In this study, serum and salivary levels of urea, creatinine, and uric acid before hemodialysis were significantly higher in CKD patients than in healthy controls. The same finding has been reported in studies that examined these markers before hemodialysis [2][13][14][15][16]. Following hemodialysis, serum and salivary levels of urea, creatinine, and uric acid significantly decreased. These results are in agreement with the findings of Chang et al, MQ Khan et al, and Seethalakshmi et al [14][15][17].

Also, in the present study, there was a positive correlation between the serum and salivary levels of urea, creatinine, and uric acid before hemodialysis, and serum and salivary levels of creatinine after hemodialysis. The obtained results are consistent with the findings of previous studies [14][15][17][18][19][16]. However, no positive correlation was observed between the serum and salivary levels of urea and uric acid after hemodialysis, which was in line with a previous study [20].

ROC analysis was performed to evaluate the diagnostic potential of salivary urea, creatinine, and uric acid compared to their serum levels. According to the ROC curve and cut-off point, the salivary level of urea in CKD patients was calculated to be 55 mg/dL with a sensitivity of 84%, a specificity of 70%, and an AUC of 0.876. It means that individuals with salivary urea levels higher than 55 mg/dL should be further evaluated for the diagnosis of CKD.

Also, the salivary creatinine cut-off point in CKD patients was 0.5 mg/dL with 81% sensitivity, 94% specificity, and an AUC of 0.881. This finding indicated that individuals with salivary creatinine above 0.5 mg/dL should be further evaluated for the diagnosis of CKD. The cut-off point obtained in this study was fully consistent with the study by Lasisi et al [6]. The salivary cut-off point was not significant for uric acid.

## Conclusion

This study showed that significant changes occur in serum and salivary levels of urea, creatinine, and uric acid following hemodialysis. Therefore, saliva analysis is suggested as an alternative to serum analysis to evaluate the efficacy of hemodialysis in CKD patients, especially in the fasting state.

## Conflict of Interest

The authors declare no conflict of interest.

## AI Disclosure Statement

During the preparation of this manuscript, the authors used ChatGPT, OpenAI company for language editing, grammar improvement, and liboberry.com for reference management. After its use, the authors thoroughly reviewed, verified, and revised all AI-assisted content to ensure accuracy and originality. The authors take full responsibility for the integrity and final content of the published article.
